# Researches, Principally Relative to the Morbid and Curative Powers of the Loss of Blood

**Published:** 1831

**Authors:** 


Art. VI.—*Researches, principally relative to the Morbid and Curative Powers of the Loss of Blood.* By Marshall Hall, M. D., F. R. S., &c. &c.—Philadelphia: Carey & Hart. 1830. pp. 173.

The subject discussed in this little work possesses a very great degree of interest to the practical physician, and cannot be too emphatically urged on the attention of the profession.

Without doubt, blood-letting is one of our most efficient and valuable curative means. Its influence is pervading and powerful, and when judiciously directed, capable of effecting the most salutary changes. It is, however, equally certain, that it is also peculiarly susceptible of great and ruinous abuse; and whilst we admit the vast benefit which it is capable of procuring, we are persuaded that there is not a remedy employed in medicine, which has been the source of so much serious and often irremediable mischief as the present one.

Much of this peculiar liability of blood-letting, to injurious consequences, arises, in a great degree, from the general inattention of physicians to the remote consequences of "loss of blood;" and still more, perhaps, to the neglect of studying the influence which various forms of disease, as well as idiosyncracy, constitutional habit, occupation, age, and other similar circumstances have, in modifying, not only the immediate, but the ulterior effects of sanguineous depletion.

The work before us is intended to illustrate all these important subjects: and we feel convinced that every practitioner, whether old or young, well may find advantage in the careful study of its contents.

The author's principal object, is to impress upon the attention of medical practitioners, the fact, that very unexpected and alarming consequences often arise from loss of blood, the nature of which is very liable to be misunderstood, and which, from being wrongly interpreted, may be readily increased to a fatal violence. He has also directed his attention especially to the illustration of "that remarkable difference in the degree of tolerance and intolerance in the loss of blood," which is found to exist in different individuals; and finally, he has endeavored to establish a distinction between "two classes of morbid affection—that of inflammations, and that of irritations." His observations on the dangerous consequences of an undue use of the lancet, appear to us highly valuable, and cannot be too strongly recommended to the attention of practitioners.

In the introductory chapter, the author illustrates the great importance of attending to the morbid effects of loss of blood, and expresses his conviction that, hitherto these topics have not sufficiently engaged "the attention of either the physiologist or of the practical physician."

"To the physiologist, the phenomena of syncope, of reaction and of sinking, present innumerable objects for his consideration of the very deepest interest. The influence of syncope on the functions of the brain, of the heart, of the capillary vessels, of the lungs, of the stomach, &c.; the phenomena of reaction, excessive or defective; but especially the phenomena and influence of the sinking state, or state of failure and decline of the vital powers, in their relation both to the nervous, the circulating, and the organic systems, severally present objects for our investigation, in a physiological point of view, at once of much novelty and the highest utility. To the physician the symptoms of reaction, so similar to those of some inflammatory affections of the head and of the heart, and the phenomena of the sinking state so similar to those of some other affections of the head, and to those of some morbid affections within the chest and abdomen, present subjects for his observation of the utmost moment in actual practice.—The diagnosis of these cases is most important, the prognosis and treatment alike, depend on it."

The effects of loss of blood, are divided into *immediate*, and *remote*. The latter, so important in their consequences, have not been sufficiently observed and investigated by the profession. They consist in the states of excessive reaction, of defective reaction, of the gradual failure of the vital powers, and of the more rapid and sudden sinking of the energies of the system, or dissolution. The phenomena of excessive reaction which sometimes result from great sanguineous depletion, may be readily mistaken by the inexperienced for an actual increased power and energy of the system, "and lead to an erroneous and dangerous employment or repetition of the lancet, when a directly opposite mode of treatment is indispensible." On the other hand, the state of actual and protracted sinking, frequently produces a series of phenomena closely resembling those which arise from sanguineous compression of the brain, or of congestion of the lungs, "so as to prompt the unwary practitioner to a still more suddenly fatal use of the lancet." These and other points relating to the excessive loss of blood, are fully illustrated in this work, and the various facts and observations which it contains cannot fail to render the practice and diagnosis in cases of this kind, much less difficult and uncertain than it must necessarily be where the knowledge which this work inculcates is not possessed.

The second chapter of the work is devoted to a cursory statement of the phenomena which attend the immediate effects of loss of blood. The most common and auspicious of these effects is *syncope.* In the occurrence of fainting, the brain appears to be the organ which first suffers functional inactivity, and this impairment of cerebral function, would seem to be the direct result of deficient sanguineous circulation in the encephalon.—From the defect of the nervous influence which is necessarily induced by this inactive state of the brain, the respiratory process becomes diminished; and the heart, both from deficient quantity of blood, and deficient decarbonization of this fluid, becomes enfeebled and more or less impeded in its actions.

That the most material circumstance in the production of syncope, is deficient circulation of blood in the brain, is evident from the effect, which all causes that promote the flow of blood in the brain, have in preventing or removing fainting. If, when syncope from loss of blood, is about to supervene, the patient suddenly assumes the recumbent posture, with the head very low, so as to favor the flow of blood to the head, and impede its rapid return, the occurrence of fainting will be prevented, or its continuation arrested if it has already taken place. Vomiting, and strong and sudden emotions of mind, as anger, by determining the blood to the head, are attended with the same result.

The tendency to syncope from loss of blood, varies exceedingly in different individuals. Some will bear the loss of an extraordinary quantity of blood without fainting, whilst others will fall into this state from the abstraction of but a very small portion of blood. This difference in the power of bearing sanguineous depletion, does not appear to depend on a corresponding difference in the constitutional powers of the system; for we frequently observe very robust and vigorous individuals, who are incapable of bearing even ordinary abstractions of blood, without falling into a syncope; whilst on the other hand, many persons of a weak and delicate habit of body, bear the loss of very considerable quantities of blood without any tendency to syncope.

*Convulsions,* also, is one of the common effects of loss of blood. This, like syncope, is manifestly the consequence of disturbed cerebral excitement. The states of inanition or exhaustion, and of repletion or fullness of the vascular system, are alike favorable to the occurrence of convulsions. Infants are much more apt to become convulsed from excessive loss of blood than adults or old people. "Convulsions occurring from blood-letting," says the author, "must be generally considered as denoting that the remedy has been used beyond the safe degree." There can be no doubt, that convulsions in the puerperal state, occasionally depend immediately on excessive sanguineous depletion; and it is particularly important, as the author observes, to distinguish these cases from those which arise from intestinal or uterine irritation.

*Delirium* is another of the immediate effects of loss of blood. Complete and protracted syncope, however, perhaps always precedes the occurrence of delirium; and this latter affection appears to be the result of inordinate reaction, and determination of blood to the brain, and not of vascular inanition in this organ. It is of the utmost importance to distinguish the delirium which sometimes occurs after excessive loss of blood, from that which arises from high febrile reaction, unaccompanied with general sanguineous inanition. The author relates several remarkable instances of alarming delirium, brought on by inordinate abstractions of blood, and preceded by deep and protracted syncope. In delirium from loss of blood, anodyne and stimulating remedies, with revulsive applications to the feet, constitute the appropriate means. In a violent case of delirium from loss of blood, the author administered fifty drops of laudanum with a drachm of spiritus ammoniæ aromaticus, with immediate good effect. In four hours after this dose was given, the delirium had subsided; but the patient remained in a state of obstinate silence, refusing to answer questions, and the pulse continued to beat 120 strokes in a minute. An ounce of brandy was then given every hour, with beef tea. "The first dose of brandy produced sleep;" and under the use of this and other supporting remedies, the patient speedily recovered.

*Coma.* Deep coma occasionally comes on as an immediate consequence of excessive loss of blood. "We may be called to patients," says the author, "so perfectly comatose, immediately after blood-letting or hemorrhage, that we may be in doubt, for a time, whether the case be not apoplexy. The history of the case—the state of the countenance—of the pulse and of the extremities, and the other symptoms will, after a little watching, make the case clear to us." We recollect a boy about 14 years of age, whom we found in a state of profound apoplectic stupor, or rather coma. He had lost a great deal of blood by hæmorrhage from the nose, the day and night previous to the occurrence of the coma. His countenance was pale, the pulse frequent and extremely small, and the extremities cold. Synapisms were applied to the feet: a stimulating enema administered. In the course of an hour he recovered sufficiently to enable him to swallow fluid. The carbonate of ammonia and wine-whey were now administered, and under these stimulants, and the use of nourishing broths, he gradually recovered.

*Sudden dissolution.* The author relates an interesting instance of sudden dissolution from inordinate abstractions of blood. The patient was robust and muscular. He had fallen from a scaffold, and fractured several ribs. Symptoms of thoracic inflammation and pulmonary lesion soon came on. He was at first bled to the extent of 18 ounces; in six hours afterwards twenty ounces more were drawn, and a purge administered: the blood was inflammatory. Next morning was bled again to the extent of 20 ounces. At noon of the same day 20 ounces of blood more were drawn; next morning 18 ounces of blood were again ordered to be drawn. "The bleeder, however, perceiving what effect even the loss of a few ounces had, desisted from drawing any more. About two hours subseqently, Mr. Lawrence saw the patient, and concurred with Mr. Lloyd as to the propriety of the further abstraction of blood; they therefore directed 20 ounces of blood more to be drawn. The pulse after this became a mere flutter, and this man only survived a few hours."

Physicians generally, do not appear to pay sufficient attention to the fatal consequences which are apt to result from copious abstractions of blood, repeated at short intervals. In subjects of an irritable habit, two or three copious abstractions of blood, sometimes gives rise to the reaction of inanition. If this condition is misunderstood, more and more blood is apt to be abstracted, without effecting the desired reduction of the arterial reaction, until the vital powers suddenly sink to a point of prostration from which the most potent stimuli cannot raise them.

In the third chapter, the author treats of *the more remote effects of the loss of blood;* and the first of these remote effects attended to is—

*Exhaustion, with excessive reaction.* This is decidedly the most important and interesting subject in relation to the morbid consequences of excessive loss of blood. "Exhaustion" says the author, "may assume several different characters. It may be attended with excessive or with defective reaction, or with actual sinking of the vital powers. The reaction or recovery from ordinary syncope is generally a simple return to a healthy state of the functions, or nearly so, the pulse not passing beyond its natural frequency. In cases of profuse loss of blood, on the contrary, the recovery is not quite so uniform, and the pulse acquires and retains a morbid frequency for a certain length of time; this frequency of the pulse may gradually subside, however, and be unattended by any other symptom of indisposition of any consequence. The phenomena are very different, if, instead of one full bleeding to syncope, or a profuse hæmorrhage, and even protracted syncope, the person be subjected to repeated blood-lettings, or to a continued drain. In this case, within certain limits, the pulse instead of being slow and feeble, acquires a morbid frequency, and a throbbing heart, and there are in some instances all the symptoms of excessive reaction.— This state of reaction is formed gradually, and consists at first, in forcible beating of the pulse, of the carotids, and of the heart, accompanied by a sense of throbbing in the head, of palpitation of the heart, and eventually, perhaps of beating or throbbing of the scrobiculus cordis, and in the course of the aorta. This state of reaction is augmented occasionally by a turbulent dream, mental agitation or bodily exertion. At other times it is modified by a temporary faintness or syncope. There is also, sometimes, irregularity of the beat of the heart and of the pulse."

In persons of an irritable and nervous habit of body, the symptoms of reaction from sanguineous inanition, sometimes become extremely vehement and alarming. A deep, throbbing pain in the head, with excessive sensibility of the sensorial nerves, and irritability of the brain, are apt, in such habits, to be added to the phenomena already mentioned. Great intolerance of light and sound occurs in some cases of this kind; "the sleep is agitated and disturbed by fearful dreams, and the patient is liable to awake or to be awoke in a state of great hurry of mind, sometimes almost approaching to delirium; often there are great noises in the head, as of singing, of crackers, of a storm, or of a cataract; and in some instances there is a sense of great pressure or tightness in one part or round the head, as if the skull were pressed by an iron nail or bound by an iron hoop."—The sense of approaching syncope, occasionally takes the place, for a short time, of the palpitation and throbbing of the heart. In awaking from sleep the patient is apt to experience "an overwhelming feeling of dissolution," or violent and agitated beating of the heart, with great anxiety and general agitation. Attacks of difficult and hurried breathing are apt to supervene, particularly on the occurrence of some mental emotion, or unusual corporeal exertion. "In this state of exhaustion, sudden dissolution has sometimes been the immediate consequence of muscular effort on the part of the patient, or of his being too suddenly raised from the recumbent into the erect position." We have thus given a full view of the author's very lucid description of the symptoms which sometimes attend exhaustion and loss of blood. The author relates several interesting examples of the phenomena of reaction, from a continued drain or loss of blood. The following case came under our own observation. A lady of a strong and robust frame of body, suffered very profuse hæmorrhage immediately after delivery. The loss of blood brought on a deep and protracted state of syncope, from which she recovered slowly. In three days after, symptoms of peritonites supervened, and the practitioner in attendance, bled her to the extent of producing signs of approaching syncope; and administered an active purge. On the following morning, there was still considerable abdominal tenderness, with frequency and sharpness of the pulse, (as I was afterwards informed.) Twenty leeches were then applied over the hypogastric region, and the patient seemed to be much relieved by the depletion. After this the symptoms of peritonial inflammation gave no further disturbance. In three or four days afterwards, however, the patient was suddenly seized with violent pain in the head, with a sense of beating within the brain, and strange and distressing noises in the ears. At times, these cephalic symptoms would suddenly cease, and a feeling of sinking or faintness supervene, with extreme anxiety in the pit of the stomach; and these spells were generally followed by vehement and tumultuous beating of the heart. In this state I was called to see her. In addition to the symptoms just mentioned, I found her pulse frequent, rather full, with a peculiar jerking beat, and so compressible as to give a sensation to the touch, as if the artery were filled with air. The countenance was extremely pale, and the prolabia in appearance wholly exanguious. The feet and ankles were slightly œdematous; and there was great muscular prostration. An attempt to assume the sitting posture, during my presence, brought on a disposition to vomit and faintness, which was succeeded by palpitation of the heart, hurried respiration, and great aggravation of the disturbance in the head. The pulse at this time, might readily have been mistaken, by a careless observer, as one of increased arterial energy and excitement, calling for further depletion. Instead of such a course, however, opium, volatile alkalic, wine, and nourishing liquid diet, were ordered; and under this stimulant treatment much relief was obtained in the course of twenty-four hours; and health eventually established.

The following case, similar to the one just detailed, is given by the author.

"Mrs. ——, a delicate married person, aged 24, a week or ten days after her confinement of a still born child, was seized with pain in the were part of the abdomen, extending to the liver, and other symptoms which indicated inflammation of the os-uteri, together with much constitutional disturbance. The case seems to have been mistaken in the beginning, so that it was allowed to become somewhat inveterate before the appropriate treatment was adopted. It was at this stage treated with leeches to the hypogastrium, cupping low in the groins, the hip-bath, aperient medicines, strict abstinence, &c. The benefit accruing from cupping over the sacrum was observed to be so decided, that recourse was had to this remedy twice or thrice a week. The disease seemed to be thus yielding in the most favorable manner, when the patient became suddenly, and quite unexpectedly affected with the effects of loss of blood, in a violent form. The quantity of blood taken by the cupping, had frequently been twenty ounces; and too exclusive attention had been paid to the disease, the state of the constitution not having been sufficiently watched. In this manner the patient became affected, all at once, after being cupped, with sudden and alarming syncope; she gasped, and became convulsed, and afterwards highly delirious. The admission of cold air, *and the administration of brandy*, gradually restored the patient to sensibility, but she remained extremely feeble. On the next morning, Mrs. —— was affected with extreme pain in the head, violent throbbing of the temples, slight delirium and sickness, and the sense of hearing was painfully acute. During the day, the pain in the head, the throbbing, and the intolerance of light and sound had increased so much with sickness, feverishness, and a frequent strong pulse, that it was apprehended that inflammation had taken place in the brain. The arm was actually tied up for bleeding; but the remonstrances of the patient, the history of the attack, and the recollection of some remarks on the subject of loss of blood, read some years before, happily led to the abandonment of this measure."

The patient, by an instinctive feeling of what would be beneficial to her, earnestly desired to have some brandy. It was given, and the result showed how disastrous would have been the further abstraction of blood. Under the use of mild and digestible diet, light cordials, rest and opiates, she gradually was freed of her disease.

These are highly instructive cases, and ought to be deeply impressed on the attention of the practitioner. We can scarcely doubt that the lancet has been frequently employed with fatal inattention or ignorance as to the true source of the symptoms of reaction and cerebral disturbance, which sometimes follow copious sanguineous depletion. Wherever manifestations of vehement reaction, and disturbance in the head come on soon after the loss of a great quantity of blood—or where such symptoms occur during the long continuance of a slow hæmorrhage, or very protracted menorrhogia, or hæmorrhail flux—more especially when these phenomena are attended with general prostration, and an exanguious appearance of the countenance, the utmost degree of circumspection should be used in the employment of depletory remedies. It will always be safest where a doubt may be entertained as to the true nature of the reaction, to try, in the first place, the effects of opiates and other stimulants. If the reaction be the result of inanition, more or less benefit will very speedily accrue from such remedies; should, however, the symptoms be aggravated, blood may then be confidently abstracted.

This course is the more necessary, since in cases of reaction from exhaustion, the loss of even a few ounces of blood may prove fatal; and every possible precaution ought therefore be used to avoid making a mistake in this respect.

We recollect an instance of a person whose system had become extremely drained and exhausted by a very protracted hæmorrhage from piles. His countenance appeared as if there was not a drop of blood flowing in his veins. In this state he was seized with great head-ache, frequent palpitations of the heart, and disagreeable inflammation of the conjunctiva. He applied to us for advice, and we ordered him the phosphate of iron, with infusion of colomba. He did not, however, follow our prescription, and to relieve the disturbance of his head, he, of his own accord, and without our knowledge, had himself bled. About four or five ounces of blood were drawn, which brought on a most alarming and protracted syncope. On the following morning he died suddenly, while attempting to raise himself in bed.

Under the head of *Exhaustion, with sinking*, the author has introduced many very valuable and interesting facts and observations.

"The symptoms of exhaustion *with excessive reaction*, may gradually subside and leave the patient feeble, but with returning health; *or they may yield to the state of sinking*, of positive and progressive failure of the vital powers, attended with a set of phenomena very different from those of exhaustion with reaction." In this impaired or sinking state, the brain becomes inactive, as is manifested by obvious sensorial torpor, a tendency to dozing and stupor, and a more or less rapid supervention of a comatose condition, with heavy snoring, stertor, "and blowing up the cheeks in breathing" while sleeping. When roused from sleep, the patient appears confused, as if stunned, and "requires a moment to recollect himself, to recover his consciousness"—or he may manifest slight and transient delirium, or gazing with an indifferent stare on the objects around him for a moment, relapse again into a state of dozing. In the majority of cases of sinking after excessive reaction, from sanguineous inanition, one of the first signs of the approaching failure of the vital energies, is a peculiar "crepitus in the respiration, only to be heard at first, on the most attentive listening; it gradually becomes more audible, and passes into slight ruttling, heard in the situation of the bronchia and trachea." This is usually accompanied with manifest labor, oppression and hurry of the respiration. The alimentary canal becomes disturbed by distention from flatus," "and the command over the sphincters is impaired." When the sinking has become extremely great, the countenance assumes a cadaveric or death-like appearance, and the patient manifests great restlessness, continually changing the position of his extremities, which are cold and insensible. In this extreme state of exhaustion, a moderate degree of delirium is almost always present. Some very instructive cases illustrative of this sinking after reaction, from loss of blood, are detailed by the author.

The author observes, that "it would, perhaps, be difficult to offer any observations on the nature and cause of *excessive reaction* from loss of blood; but it is plain that the state of sinking involves a greatly impaired state of the functions of all the vital organs, and especially of the brain, *from defective stimulus*." It appears to us, however, that the nature and cause of excessive reaction from loss of blood, is not so inexplicable as the author seems to think. We are inclined to believe, that in cases of inordinate reaction from loss of blood, there exists, in fact, no vascular inanition; but on the contrary, actual repletion; and our reasons for this opinion are the following circumstances. The reaction in cases of this kind, never comes on until several days after the excessive loss of blood; and it is, indeed, chiefly in instances where there is slow and protracted hæmorrhage that the symptoms of reaction, at last supervene. Now, when we advert to the fact, that absorption is increased in proportion as the blood-vessels are deprived of their contents, we may conclude, that where by hæmorrhage or the too free use of the lancet, the blood-vessels are, in a considerable degree, deprived of their contents, absorption will be greatly accelerated, in consequence of which, the blood-vessels will be replenished by a crude and watery fluid. As the abstraction or diminution of the habitual stimulus of the blood, must at the same time have the effect of greatly augmenting the excitability of the system, we have thus all the conditions most favorable to the production of inordinate reaction—namely, a crude and irritating fluid circulating in the blood-vessels, and augmented susceptibility to the impressions of the fluid. That the blood in such cases is in the condition we have stated, is demonstrated by its appearance. It always contains a vast proportion of serum, the coagulable part being usually very small. But to return to our author.

*Exhaustion with delirium* is considered in the fourth section of this chapter. The author quotes an interesting instance of this kind from Dr. Abercrombie. A lady a fortnight after a delivery of her fifth child, was seized "with pain and deep-seated hardness in the right side of the pelvis—very tender to the touch, and accompanied by a considerable degree of fever."—By repeated topical bleeding, the disease was apparently subdued; her strength, however, remained much reduced, so that, though free from pain or fever, she was still confined to bed four weeks after the beginning of the disease. In this weak and exhausted state, "some family occurrence of a trifling nature," greatly agitated her mind. On the following morning her brain was in a state of extraordinary excitement. She talked incessantly, and screamed and struggled wildly; her pulse was small and rapid. *Leeches* were applied to the temples, and cold to the head, and a purgative given. The symptoms were aggravated —her pulse became still more rapid and feeble, and her countenance manifested great exhaustion. The stimulant plan was now adopted; "a full glass of wine was accordingly given, and ordered to be repeated every hour." In four hours after the first dose of wine, the patient "was composed and rational—the pulse was 90, and of good strength, and from this time there was no return of the symptoms."

"I am persuaded," says the author, "that excessive loss of blood is by far the most frequent and influential source of delirium or mania, occurring in the puerperal state." Our own experience has led us to believe that, in some instances at least, this form of mania arises from the cause mentioned by the author. About four years ago, we attended a lady in her first accouchment. A portion of the placenta was attached over the mouth of the uterus, and she lost a most alarming quantity of blood during the delivery, which caused protracted syncope.—She did pretty well, however, for five or six days, though greatly exhausted. At the end of this time she began to talk incoherently, complaining, at intervals, of a distressing sensation in the head. In the course of twenty-four hours, a complete state of mania was developed. The pulse was very frequent, jerking, and somewhat small. As she had lost a great deal of blood only five or six days previous, we did not venture to use the lancet, but 20 leeches were applied to the temples, and an active mercurial purgative prescribed. In twelve hours after this, all the symptoms of the disease were very conspicuously aggravated. Full doses of camphor and hyoscyamus were now ordered, and the result was singularly favourable. Ten grs. of camphor, with four of the narcotic, were ordered every four hours. Before the time for giving the third dose arrived, the patient was tranquil, and manifested but very slight mental aberration. By the continued use of smaller doses of this mixture, and aperients, the disease was entirely removed in the course of four or five days.

*Exhaustion with coma* is but rarely met with, except in the last stage of sinking, from sanguineous inanition. In infants and children, however, we occasionally meet with a comatose insensibility in connection with great exhaustion from loss of blood.

Dr. Abercrombie makes the following observations on this point:

"In the last stage of diseases of exhaustion, patients frequently fall into a state resembling coma, a considerable time before death, and while the pulse can still be felt distinctly; and I have many times seen children lie a day or two in this kind of stupor, and recover under the use of wine and nourishment. It is often scarcely to be distinguished from the coma, which accompanies diseases of the brain. It attacks them after some continuance of exhausting diseases, such as tedious and neglected diarrhœa; the patients lie in a state of insensibility, the pupils dilated, the eyes open and insensible, the face pale, and the pulse feeble. It may continue for a day or two, and terminate favorably, or it may prove fatal. This affection appears to correspond with the *apoplexia ex inanitione* of the older writers. It is produced by causes that exhaust gradually, and not, like syncope, by sudden and temporary causes of exhaustion. I have seen the same affection in adults: a man considerably advanced in life, in consequence of exhaustion from a neglected diarrhœa, fell into a state closely resembling coma. He was recovered from this state by the use of wine and opiates."

Upwards of 20 years ago, we were called to an old man, a farmer, whom we found in a state of comatose insensibility—with a pale and shrunken countenance, small and feeble pulse, and cool skin. We were informed, that for six weeks previous, and up to the time when he sunk into the coma, he was much harassed by diarrhœa, and that for the last ten days he had taken but little nourishment. He was naturally a man of a robust and vigorous habit of body, and until he became affected with diarrhœa, remarkably active for his age. In the small and feeble state of his pulse, bleeding, whether local or general, was out of the question; and, in truth, from our inexperience at that time, we were extremely embarrassed as to the course we should pursue. We ordered him sinapisms to the feet, frictions of the body and extremities with some stimulating liximent, and a stimulating injection. With this direction we left him, expecting to find him dead at our next visit. In the meantime, his friends, who observed some amendment from the use of the means just mentioned, gave him, from time to time, considerable portions of wine, which had the effect of entirely removing the comatose affection in the course of eight or nine hours.

*Exhaustion* from excessive loss of blood, sometimes gives rise to torpor or paralysis of the optic nerve, causing more or less complete blindness or *amaurosis*. Mr. Travers has made some interesting observations on this subject, in his work on the Diseases of the Eye, and to which the reader is referred, for a detail of the circumstances attending these cases.

*In the fourth chapter* the author treats of "the effects of further loss of blood, in cases of exhaustion."

"The symptoms of exhaustion," says the author, "with reaction have, I am persuaded, frequently been mistaken for those of inflammation, or other disease of the head, or of the heart.—Under this impression, recourse has been had to the further detraction of blood by the lancet. And the effect of this practice is such as greatly to impose upon the inexperienced; for all the symptoms are, perhaps, greatly relieved. It was sometime before I could fully comprehend the nature of this fact. I had satisfied myself that in certain cases, the symptoms were those of loss of blood; and yet it appeared no less certain, that those very symptoms were relieved by the lancet. At length I discovered, by a careful observation, that the symptoms which were relieved, were those of reaction, and that the mode of relief *was by the substitution of syncope*; that the relieve endured as long as the state of faintness continued, but returned as this state gave way to the rallying and reaction of the vital powers. Another circumstance equally interesting and curious was, that within certain limits, the remedy which relieved for a time, eventually only added to the severity of the malady, for this was apt to return after a certain period, in a still more aggravated form." In exhaustion attended with reaction, the loss of a few ounces of blood, is in general, sufficient to cause syncope. During this state, and for a few hours afterwards, the symptoms of reaction are usually allayed, and though much prostrated, the patient feels better. The reaction, however, soon returns, generally with renewed vehemence; and as the system is still more exhausted by the additional loss of blood, all the dangerous conditions of the case are greatly enhanced.

It is not uncommon to meet with instances like this: a person, after copious and protracted hæmorrhage, is seized with violent beating, and pain in the head, a rushing sound in the ears, great intolerance of light and sound, a frequent and active pulse, and warm skin. Blood-letting *seems* to be indicated—the arm is tied up, and the vein opened. After the loss of a few ounces of blood, the patient *faints*, and on recovery from this state, he feels much relieved, all the symptoms of cerebral disturbance being allayed. In three or four hours, however, the former symptoms of reaction return, and as relief was obtained from the use of the lancet before, a vein is again opened. The blood flows for a short time—the patient again faints, but now the vital powers are either suddenly extinguished, or a slight degree of reaction returns, from which the patient more or less rapidly sinks into the embraces of death.

"When the last bleeding has been considerable, it has, in some cases, been followed by the most dreadful gaspings, and other convulsive motions of death. It should be observed, that between the most gradual sinking, and the most sudden dissolution, as the effects of blood-letting, there is every intermediate shade, with the phenomena, of which it is of the utmost importance to be acquainted." The author has given a very interesting case illustrative of these varied phenomena. Mrs. ——, aged 30, was affected with slight influenza, during this disease; labour came on, which issued in delivery in about 15 hours. This was succeeded by slight fever, attended with "incessant cough." Forty ounces of blood were drawn during the first 24 hours, besides what was drawn by twelve leeches. On the next day, 12 ounces more of blood were drawn, and during the flow of the blood, the patient became faint, and sank to a state of exhaustion from which she never again rallied. "She became extremely feeble, and could scarcely articulate; and during the three succeeding days, there was a state of extreme exhaustion—and still a sense of load at the chest and pain of the side."

At the end of this period, the countenance was observed to change frequently, from a deep flush to great paleness—the perspiration ran down her face—the pulse was extremely frequent, with severe pain on coughing: no delirium, but agitated and hurried waking from sleep, and "distressing faintings usually came on about two or three o'clock, P. M." In five or six days after the supervention of these symptoms, "she became drowsy, and evidently more sinking"—and this state continued to increase until in the course of ten or twelve hours, the patient died. "The last blood-letting in this case," says the author, "was obviously though rather gradually fatal." A case is next detailed, in which "the fatal effect supervened immediately on the use of the lancet." We once met with an instance of this kind. The patient, a female, had lost a great deal of blood during and immediately after parturition. In three weeks after her confinement, she was anasurcous, her countenance extremely pale; the lips appearing wholly bloodless. The slightest motion or exertion to turn in bed, caused palpitation of the heart, and great disturbance in the head; and every attempt to raise the head from the pillow, caused sickness at the stomach, with a sense of approaching syncope. Notwithstanding these manifestations of exhaustion, the pulse was frequent and active. The attending physician, very young and inexperienced, regarded the state of the pulse as indicating the propriety of blood-letting. The arm was tied, the patient remaining in the horizontal posture, and about eight ounces of blood drawn, without the supervention of syncope. In about five minutes, however, after the blood was drawn, the patient suddenly turned on her back, gasped violently for a few moments, and expired.

*Chapter V*, is occupied with the consideration "of the influence which various circumstances have on the effects of loss of blood." As this subject is more fully discussed in the "*Second Part*" of this work, we omit here, entering into an analysis of the various topics which it embraces. We may observe, however, that the author does not appear to us to dwell with sufficient emphasis and earnestness on *intestinal irritation*, as a condition capable of modifying the effects of loss of blood. This is a most important subject. Intestinal irritation exerts, often, the most extraordinary influence upon the powers of the system to sustain the loss of blood. We do not here refer to vascular or capillary irritation—a condition which approaches to the state of inflammation, but to that peculiar impression and excitement in the intestinal nerves, which certain offensive or irritating substances lodged in the alimentary canal are capable of producing.

It is perfectly well known, that the impression of irritating substances on the intestinal nerves, often causes a general nervous and vascular torpor and depression, altogether similar to the prostration which sometimes arises from concussion, or violent comminuted fracture of an extremity. This state of constitutional irritation from local injuries, and intestinal irritation, has been abundantly illustrated by Travers, Armstrong, and the author of the present work. In febrile diseases we sometimes meet with instances of unexpected failure of the vital actions, resembling true sinking, though not dependent on actual exhaustion. In such cases, the free operation of a purgative will, in general, speedily remove the symptoms of prostration, and re-establish the actions of the system. The detraction of blood in cases of this kind, never fails to increase the prostration, and may readily reduce the vital energies below the point of recovery. This constitutional depression from intestinal irritation is especially apt to occur in febrile diseases, after profuse hæmorrhage; and hence we not unfrequently meet with it in the puerperal state. Females of a nervous temperament, are most apt to suffer prostration from this cause, more especially if sufficient attention has not been paid to the due evacuation of the alimentary canal, during the latter stage of pregnancy, or soon after parturation.

From this fact, we perceive, that if symptoms of exhaustion or prostration come on some time after profuse hæmorrhage, we are not to infer at once, that it is the result, solely, of sanguineous inanition, unless, indeed, the manifestations of this condition are so decided that they cannot be mistaken. In cases which *seem* to be the direct consequence of deficient blood, the operation of a purge will sometimes do away all the alarming symptoms of prostration, by removing the sources of intestinal irritation. In order to obviate the unfavorable consequences which might ensue from the operation of a purge, when actual exhaustion is present, it will always be proper in cases, (where a doubt may exist, whether the prostration be the result of sanguineous inanition, or of intestinal irritation,) to administer stimulants, such as wine, or the carbonate of ammonia, in conjunction with the purge.

In the spring of 1829, we attended a lady in her confinement with her first child. She lost an unusual quantity of blood immediately after the birth of the child. She, nevertheless, did very well until the fourth day, when she was seized with strong chills, followed by violent fever, and much pain and tenderness in the hypogastric region. Blood was drawn to the extent of 20 ounces, a purgative adminstered, which operated well, and the nitrous powders, with calomel, prescribed. On the following day, 30 leeches were applied over the hypogastrium, and another aperient administered. By these measures she was brought to a state of convalescence by the end of the seventh day after the commencement of the fever. She continued, after this, gradually to improve, and I left off my daily attendance. About the end of the second week, I was again sent for, and I found my patient laboring under moderate febrile symptoms, attended with painful diarrhœa, great watchfulness, and a state of inquietude. The pulse was frequent, but small and tense. A mild purgative was ordered, with a diaphoretic mixture, composed of spirit. minder, vin. antim., spir. nitre, dulc and lemon syrup.

On the following morning, I found her in a very depressed condition. The extremities were cool and damp; the head confused; the pulse very small, weak and frequent; the countenance pale, attended with great anxiety, restlessness, and instead of the previous wakefulness, a very marked state of drowsiness. She also complained of a most intense pain in the heel of the left foot, although not the slightest redness or swelling could be noticed in the part; and pressure did not increase, but rather diminish her sufferings. There was at times much tormina, but very little alvine evacuation.

We were now informed that the day before this second attack came on, she was induced by a friend to eat a large piece of plum-pie. A full dose of castor oil, with two drachms of spirits of turpentine were now administered, which brought away an enormous quantity of exceedingly fetid feculent matter. Almost immediately after these evacuations, the symptoms of prostration and torpor were entirely removed, and under a careful regimen she recovered her health without any further difficulties.

When there is reason to believe that intestinal irritation cooperates with vascular inanition, in the production of exhaustion, *opiates* are, in general, more beneficial than the more diffusible stimulants.

*Chapter VI* is devoted to the consideration "of the effects of loss of blood on the internal organs." The author refers particularly to the effusion of serum into the cavities of the brain; and the singular tendency, in fatal cases, to the production of capillary engorgement or injection in the mucous membranes, as well as in the meninges of the brain. The experiments of Seeds and Kellie are well known. From these it appears that, when animals are killed by bleeding, the capillaries of the membranes of the brain always exhibit a degree of injection which might be mistaken for inflammation, attended with copious serous effusion into the ventricles.

It appears, even, that apoplexy may occur during great exhaustion, from sanguineous inanition. The author quotes a case from Dr. Denman, in which, after the profuse and protracted discharges of blood from a fungous growth of the uterus, and great consequent exhaustion, fatal apoplexy suddenly supervened. On dissection, the ventricles were found to contain about four ounces of blood. "This extreme exhaustion," says the author, "was clearly the immediate cause of her death, little as it might have been expected from the daily profuse discharge to which the patient had been subject."

The condition of the lungs, produced by excessive loss of blood, is compared, by the author, to the state of derangement caused in this viscus, by dividing the 8th pair of nerves. "Still there is a difference, for instead of hepatization with destruction of the texture of the lungs, there is œdema of this organ, with a clogged condition of the bronchia." When the hæmorrhage approaches to a fatal extent, the bronchia and air cells first become surcharged with mucous; more or less œdematous effusion then occurs into the pulmonary tissue, and finally, deficient arterealization from both these causes is superadded to prostrate the system, and extinguish the spark of life. In two cases of fatal hæmorrhage reported by Dr. Hodgkin, the lungs were very decidedly œdematous. These conditions will account for the great pectoral oppression and difficulty of breathing, which generally occur some time before death in slow hæmorrhages.

*In Chapter VII*, the author speaks "*of the treatment* of the various effects of loss of blood." The reader need scarcely be informed, that stimulant, anodyne, and invigorating means, are the only appropriate remedies for the various affections in question. "Syncope, reaction, and sinking, each requires its appropriate treatment. The constitutional treatment must be stimulant in syncope; anodyne or sedative in the state of reaction; and invigorating or restorative in that of sinking. When syncope assumes a dangerous form, the principal remedies are, an attention to the posture of the patient, which should be horizontal or even with the head somewhat depending, stimulants, both external and internal, and the transfusion of blood." In cases of excessive reaction from exhaustion, the appropriate means are, perfect quietude, both of body and mind—small doses of opium, or what the author prefers, hyosyamus, mild and liquid nourishment. "The tincture of hyosyamus," says Dr. Hall, "is, I think, the kindliest anodyne and sedative in these cases." Very minute portions of brandy may be added to the mild nutriment; but the use of diffusible stimulants in cases of this kind requires the utmost degree of circumspection, and they can never be used with propriety except in very small doses. "It may be necessary to subdue the throbbing and pain in the head, by local blood-letting; and it is most remarkable how small a quantity of blood taken will relieve. Two or three leeches are frequently quite sufficient."

In the second appendix to the first part of this work, the author has given a very interesting account of "*A hydrencephaloid affection of infants arising from exhaustion*," and although we have already extended this article to a considerable length, we think we shall perform an acceptable service to our readers, in presenting them an abstract of this chapter.

There is a source of disorder, in infancy, less frequent, perhaps, in its operation, than the irritation of teething, and gastro-intestinal irritation and inflammation, but not less important in its consequences, and far less understood by medical men—namely, *exhaustion*. "This exhaustion has its origin, during early infancy, chiefly in diarrhœa or catharsis; in the latter periods of infancy, in the loss of blood, with or without the relaxed or diarrhœal condition of the bowels." The diarrhœa is generally caused by improper food; and the excessive catharsis is often the consequence of the too frequent administration of harsh purgatives. "The exhaustion from loss of blood usually follows the inappropriate or undue application of leeches or use of the lancet." The author declares it as his opinion, that the majority of fatal diseases during infancy, "occur from this inappropriate or undue application of exhausting remedies;" and although we are not disposed to go to this extent with our author, we are nevertheless fully satisfied, from long and close observation, that a vast amount of suffering and fatality in infancy, arises from the cause indicated by the author.

The infantile disease to which the author has more especially directed his attention, is what he denominates *hydencephaloid disease*. Its course is divided into two periods or stages. The first is characterized by morbid or excessive irritability; the second by the reverse condition of torpor.

"This morbid affection, as I have already stated, has usually been first induced by some change in diet, by which the stomach has been loaded or disordered, and the bowels, perhaps, affected with diarrhœa; and this latter state has frequently been exasperated by the untimely administration of an aperient medicine. The infant becomes irritable, restless and feverish; the face flushed, the surface hot, and the pulse frequent; there is an undue sensitiveness of the nerves of feeling, and the little patient starts on being touched, or from any sudden noise; there are sighing moaning during sleep, and screaming; the bowels are flatulent and loose, and the evacuations are mucous and disordered."

If through an erroneous notion, as to the nature of this affection, nourishments and cordials are not given; or if the diarrhœa continue, either spontaneously or from the administration of medicine, the exhaustion which ensues is apt to lead to a very different train of symptoms. The countenance becomes pale, and the cheeks cool or cold; the eye-lids are half closed, the eyes are unfixed, and unattracted by any object placed before them, the pupils unmovod on the approach of light; the breathing from being quick, becomes irregular and affected by sighs; the voice becomes husky, and there is sometimes a husky, teasing cough; and eventually, if the strength of the little patient continue to decline, there is crepitus or rattling in the breathing; the evacuations are usually green, and the feet are apt to be cold. During the state of irritability, the breathing is quick; during that of the torpor, it is slower, irregular, suspirious, and finally crepitous. The author observes, that nothing is so characteristic of exhaustion in infants, as "pallor and coldness of the cheeks, with half-closed eye-lids, and irregular breathing."

Dr. Abercrombie refers to this affection of infants in the following words:

"In the last stage of diseases of exhaustion, patients frequently fall into a state resembling coma, a considerable time before death, and while the pulse can still be felt. It attacks them after some continuance of exhausting diseases, such as tedious or neglected diarrhœa; and the patients lie in a state of insensibility, the pupils dilated, the eyes open and insensible, the face pale, and the pulse feeble. I have many times seen children to be a day or two in this kind of stupor, and recover under the use of wine and nourishment."

The remedial treatment of cases of this kind, consist of means calculated to restore the healthy action of the bowels, and to support and invigorate the general energies of the system. If diarrhœa be present, minute doses of calomel, in union with prepared chalk, with an occasional small dose of tinctura opii, will, in general, suffice to remove this source of general exhaustion and disorder. To support and strengthen the system, the carbonate of ammonia, wine, brandy, and proper nourishment may be deemed indispensable. But "the young milk of a young and healthy nurse, is the remedy of most importance—in the absence of which ass's milk may be tried." Considerable benefit may be derived from stimulating frictions on the extremities; and a small blister or sinapism on the nape of the neck, will, in general, assist materially in removing the comatose stupor. "It is also of the utmost importance carefully to avoid putting the little patient in the erect position." We conclude this article by extracting from the work before us, the following case, illustrative of the affection just described.

"A little girl, aged four months, was seized with a bowel complaint; the usual medical attendant prescribed an aperient, which acted too freely. When I saw it on the second or third day of the disorder, the countenance was pale and sunk, and the cheeks cool; it started on being touched; there was a peculiar huskiness of the voice, and the pulse beat from 144 to 150. By giving brandy, the pulse was found on the succeeding day, reduced to 120, and there was some apparent amendment, although a degree of ruttling in the breathing or on coughing, was now added to the huskiness of the voice. By continuing the brandy, the cheeks became warm, and at length somewhat flushed, and the pulse rose to 140. The quantity of brandy was diminished and cautiously regulated, and the pulse very gradually fell to the natural standard.

"In this case the pallidness and coldness of the cheeks, and the state of the voice and breathing, indicated almost a fatal degree of exhaustion; the frequency of the pulse, arising from this cause, was reduced by the brandy; but it was afterwards again increased as the effect, not of exhaustion, but of the stimulus, and the cheeks recovered their warmth, and sometimes even became flushed. In another case precisely similar, the state of sinking continued in spite of every remedy, and the little infant lingered, and then expired.—I have known such a state of lingering to be continued for several days."

We have thus given a very full account of the contents of the *first part* of Dr. Hall's Book. The length to which this article has already been extended, prevents us, in this place, from entering upon an analysis of the *second part* of the work. It contains, however, many highly interesting observations "on the curative effects of loss of blood"—a subject to which we intend to devote some pages of a future number of this Journal.

